# Exclusive breastfeeding practices among mothers in urban slum settlements: pooled analysis from three prospective birth cohort studies in South India

**DOI:** 10.1186/s13006-017-0127-8

**Published:** 2017-08-01

**Authors:** Vasanthakumar Velusamy, Prasanna S. Premkumar, Gagandeep Kang

**Affiliations:** Wellcome Trust Research Laboratory, Division of Gastrointestinal Sciences, Christian Medical College, Vellore, -632004 India

**Keywords:** Prospective study, Exclusive breastfeeding, Sociodemographic factors, Urban slums, India

## Abstract

**Background:**

The World Health Organization (WHO) recommends six months of exclusive breastfeeding. Despite documented health, social and economic benefits, the practice of exclusive breastfeeding is quite low and information on influencing factors is limited especially from slum settlements. Our goal is to assess the prevalence and evaluate factors associated with early cessation of exclusive breastfeeding in the first six months of life among mothers in urban slums of Vellore, Southern India.

**Methods:**

We pooled data from three similar birth cohort studies (*n* = 1088) conducted between 2002 and 2009. Breastfeeding information was obtained soon after birth and then from follow-up home visits conducted once every two weeks by the field workers. Multivariable Cox regression analyses were used to assess factors associated with early cessation of exclusive breastfeeding.

**Results:**

The prevalence of exclusive breastfeeding for the first six months was 11.4%, based on prospective data since birth. Results from multivariable analyses revealed maternal education (Adjusted Hazard Ratio [AHR] 1.18 , 95% CI 1.03, 1.35), pucca type of house (AHR 1.25 , 95% CI 1.10, 1.43), two or more number of children in the family (AHR 1.26 , 95% CI 1.10, 1.43), joint family structure (AHR 1.20 , 95% CI 1.02, 1.40) and birth during summer (AHR 1.16, 95% CI 1.01, 1.31) were associated with early cessation of exclusive breastfeeding in the first six months.

**Conclusions:**

Our results indicate that exclusive breastfeeding rates are well below the recommended levels. Educational interventions providing comprehensive breastfeeding information to mothers and their families can be evaluated to assess its effect on improving infant feeding practices.

## Background

The importance of exclusive breastfeeding is well recognized; children who are exclusively breastfed for six months have a lower risk for gastrointestinal infections, respiratory illness, poor linear growth and cognitive impairment [1]. Moreover, it is described as one of the best cost effective interventions and the recommended infant feeding method for the first six months. Exclusive breastfeeding can prevent 823,000 annual deaths or 13.8% of all deaths of infants younger than 24 months, and the aim is that the breastfeeding coverage be scaled up to universal levels (90%) [1, 2]. Despite its demonstrated benefits, the practice of exclusive breastfeeding is not common in many developing countries including India [3]. Data from the National Family Health Survey (NFHS-III, 2005–2006) show that less than 49% of Indian mothers exclusively breastfeed their infant for the period of six months [4]. Few studies conducted in slum settlements show exclusive breastfeeding rate is further lower in these settings, ranging between 15 and 30% [5, 6].

Past studies have identified socio-economic status, maternal education, family structure, gravida, utilization of antenatal care services, place of residence and access to information as correlates of exclusive breastfeeding [7]. However, the results have been conflicting in terms of the direction and magnitude, suggesting that social context and environmental factors could play an important role. These past studies were small, cross-sectional, and do not typically report the breastfeeding practices in specific settings such as urban slums. Rapid urban economic and population growth has outpaced the ability to provide adequate infrastructure, leading to a majority of urban residents to live in poor settings. These settings are characterized by inadequate water and sanitation and a limited education, health and other social services, thereby increasing the risk for a range of diseases. Given that slums are expanding at a fast rate and will present unique public health challenges [8], identifying factors that influence exclusive breastfeeding will be important to design tailored interventions that has a greater chance of success. Using data from prospective birth cohort studies, we aim to assess the prevalence and evaluate factors associated with early cessation of exclusive breastfeeding among mothers in urban slums of Vellore, Southern India.

## Methods

Vellore is a Southern Indian city with a population of approximately 185,803 (census report, 2011) [9]. We combined data for this analysis from three community based prospective birth cohort studies conducted in urban slums of Vellore. These studies were conducted to understand the epidemiology and natural history of childhood enteric infections, including transmission patterns and the role of nutrition, growth and morbidity. The studies were launched in 2002, 2008 and 2009, with a maximum follow-up duration of three years. All three studies followed similar protocols for recruitment and data collection. Pregnant women who intend to stay in the study area for the follow-up period were recruited and followed up until delivery. Children were enrolled soon after birth, and fieldworkers visited each household to obtain baseline information on demography, socio-economic indicators, and birth details. Breastfeeding at birth was assessed during this first interview after birth. They were then followed up through home visits by the fieldworkers every two weeks from birth. At each follow up interview, mothers were asked if the child consumed only breast milk or breast milk together with other food or liquids (aside medications) since the previous interview. Once a mother reported the consumption of other foods, data collection with regard to exclusive breastfeeding was terminated. Further details on the study population and methodology of individual studies were described in detail elsewhere [10–12]. All studies were approved by appropriate institutional ethics committees. Informed consent was obtained at recruitment from mothers in the original birth cohort studies.

### Data and definitions

We defined exclusive breastfeeding according to the WHO recommendation [13]. The infant had received only breastmilk and had not been fed any other food or fluid with the exception of oral rehydration solution, drops and syrups (vitamins, minerals and medicines) during the period of 0–6 months. We determined exclusive breastfeeding status since birth using prospective assessment of breastfeeding status obtained during the follow-up visits. The independent variables of interest include maternal age (≤ 20, > 20), maternal education (yes, no), gravid (primi, multi), months of birth (wet; September–March, dry; April–August), place of birth (home, hospital), history of abortion (yes, no), mode of delivery (caesarian, vaginal), birthweight of the infant (low, normal), gender of the child (male, female), number of children (< 2, ≥ 2), number of family members (≤ 5, > 5), socio-economic status (low, middle, high), type of family (nuclear, joint, extended) and house (kutcha, mixed and pucca). The type of house was determined by considering the quality of materials used for construction of its walls and roof and divided into three categories: pucca - brick walls and concrete roof; kutcha - mud walls and thatched roof; mixed - mud walls with brick walls or thatched roof.

### Data analysis

We used Kaplan-Meier survival approach to estimate median duration of exclusive breastfeeding and rates of exclusive breastfeeding over the first six months. For this approach, cessation of exclusive breastfeeding was considered as the event of interest. Mothers who continue to exclusively breastfeed after the first six months and those lost to follow-up were considered as censored observations. Log-rank test was used to compare duration of exclusive breastfeeding for risk factors. Factors that were associated with exclusive breastfeeding with *p* < 0.1 were included in the multivariable Cox proportional regression model. The proportionality assumption was assessed using Schoenfeld residuals. Adjusted and unadjusted hazard ratios were reported with 95% confidence intervals and *p* - values*.*

## Results

### Sociodemographic characteristics

**Fig. 1:**
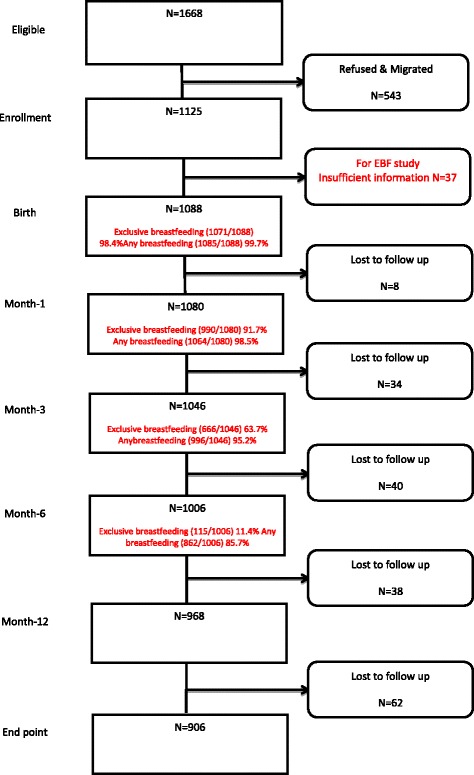
Flow chart of participants

Figure 1 shows the flow chart of the cohort. Of the initial 1125 pregnant women enrolled, 1088 (96.7%), 1080 (96%), 1046 (93%) and 1006 (89.4%) were followed up at birth, one month, three month and six month, respectively. Table 1 provides socio-demographic characteristics of the study sample. The mean age of the mothers was 23.9 (SD = 3.8) with 19% in the age group less than or equal to 20 years. One-third of mothers did not have any formal education (32.2%), and first-time mothers (33%), and 64.3% were from low socio-economic status. About 81% lived in nuclear/extended families, 84.6% had normal delivery and 97.8% delivered in health institutions.

**Table 1:** Sociodemographic characteristics

Characteristics	*n* = 1088 (%)
Mean age of the mothers (SD)	23.9 (3.8)
SES	
Low	699 (64.3)
Moderate/High	389 (35.7)
Family type	
Nuclear/Extended	882 (81)
Joint	206 (19)
Mothers education	
No formal education	350 (32.2)
Education	738 (67.8)
Parity	
Primi	359 (33)
Multi	729 (67)
Mode of delivery	
Cesarean/Instrument	167 (15.4)
Vaginal	921 (84.6)
Place of birth	
Home	24 (2.2)
Hospital	1064 (97.8)

### Breastfeeding indicators

**Fig. 2:**
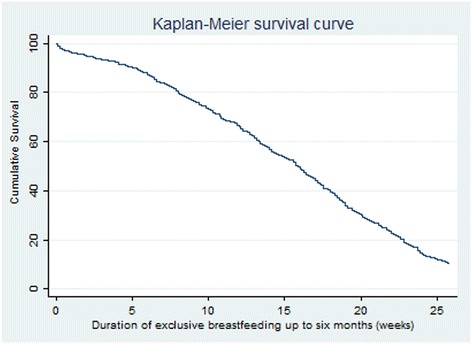
Duration of exclusive breastfeeding

Figure 2 shows the Kaplan-Meier curve for duration of exclusive breastfeeding in the first six months after birth. The median duration of exclusive breastfeeding was 15.9 weeks (approximately four months). Of the 1088 mothers, 98.4% of the mothers exclusively breastfed at birth, this declined to 91.7% and 63.7% in the first and third month respectively. By the sixth month, only 11.4% of the mothers exclusively breastfed their infants. Rates of any breastfeeding at birth, first, third and six months were 99.7%, 98.5%, 95.2% and 85.7% respectively.

### Bivariate and multivariable analyses

Bivariate analysis indicated maternal education, house type, number of children, family structure and birth season were associated with cessation of exclusive breastfeeding in the first six months (Table 2). In the multivariable analysis (Table 3), the risk of cessation was higher among mothers with an education compared to mothers with no formal education (Adjusted Hazard Ratio [AHR] 1.18, 95% CI 1.03,1.35). The number of children in a family was a significant determinant: mothers with two or more children were more likely to cease exclusive breastfeeding compared to mothers with less than two children (AHR 1.26, 95% CI 1.10, 1.43). Living in a pucca house was associated with increased risk for cessation of exclusive breastfeeding than living in kutcha or mixed house type (AHR 1.25, 95% CI 1.10, 1.43). Mothers in nuclear/extended family, compared to mothers from joint family, are more likely to cease breastfeeding (AHR 1.20, 95% CI 1.02, 1.40). Mothers who gave birth during the summer months were more like to cease exclusive breastfeeding (AHR 1.16, 95% CI 1.01, 1.31).

**Table 2:** Bi-variate analysis of factors associated with cessation of exclusive breastfeeding

Characteristics	*n* = 973	*n* = 115	*p*-value	HR	95% CI
	Non-EBF (%)	EBF (%)			
Number of children					
> = 2 children	378 (92.4)	31 (7.6)	0.005*	1.20	1.06,1.37
< 2 children	595 (87.6)	84 (12.4)			
Number of persons					
< = 5 persons	577 (88.9)	72 (11.1)	0.50	1.05	0.92,1.19
> 5 persons	396 (90.2)	43 (9.8)			
SES					
High	361 (92.8)	28 (7.2)	<0.001*	1.30	1.14,1.48
Low/Mod	612 (87.5)	87 (12.5)			
Family type					
Joint	195 (94.7)	11 (5.3)	0.02*	1.21	1.04,1.42
Nuclear/Extended	778 (88.2)	104 (11.8)			
House type					
Pucca	579 (91.9)	51 (8.1)	0.001*	1.23	1.08,1.40
Kutcha/Mixed	394 (86.0)	64 (14.0)			
Mother’s age					
> 20 years	791 (90.0)	88 (10.0)	0.32	1.08	0.92,1.27
< = 20 years	182 (87.1)	27 (12.9)			
Mother’s Education					
Education	670 (90.8)	68 (9.2)	0.008*	1.20	1.05,1.38
No formal education	303 (86.6)	47 (13.4)			
Parity					
Multi	651 (89.3)	78 (10.7)	0.75	0.98	0.86,1.12
Primi	322 (89.7)	37 (10.3)			
Month of birthing					
Summer	380 (91.8)	34 (8.2)	0.02*	1.16	1.02,1.32
Winter	593 (88.0)	81 (12.0)			
Abortion (previous)					
No	852 (90.1)	94 (9.9)	0.83	0.98	0.81,1.19
Yes	121 (85.2)	21 (14.8)			
Place of delivery					
Hospital	954 (89.6)	110 (10.4)	0.29	1.28	0.81,2.01
Home	19 (79.2)	5 (20.8)			
Mode of delivery					
Vaginal	822 (89.2)	99 (10.8)	0.11	0.87	0.73,1.03
Instrumental/Cesarean	151 (90.4)	16 (9.6)			
Low Birth Weight					
No	816 (89.7)	94 (10.3)	0.61	1.05	0.87,1.26
Yes	136 (89.5)	16 (10.4)			
Child’s sex					
Male	508 (89.8)	58 (10.2)	0.91	0.99	0.88,1.13
Female	465 (89.1)	57 (10.9)			

*Significant at 5% level; *HR* Hazard Ratio

**Table 3:** Multivariable analysis of factors associated with cessation of exclusive breastfeeding, from Cox proportional hazard analysis

Determinants	Reference	AHR	95% CI	*p*-value
No of children: > = 2 children	< children	1.26	1.10,1.43	0.001
House type: Pucca	Kutcha/Mixed	1.25	1.10,1.43	0.001
Family type: Joint	Nuclear/Extended	1.20	1.02,1.40	0.03
Mother’s education: Education	No formal education	1.18	1.03,1.35	0.02
Month of birthing: Summer	Winter	1.16	1.01,1.31	0.03

*AHR* Adjusted Hazard Ratio

## Discussion

In this study, only one-tenth of the mothers exclusively breastfed their infants for a period of six months from birth. Prospective studies from India are limited, but studies from neighbouring countries such as Sri Lanka 49.1% [14], Maldives 41% [15] and Nepal 29.7% [16] reported higher rates of exclusive breastfeeding from birth. These large differences might be due to the fact that mothers residing in slum settlements were not exposed to promotional messages and initiatives aimed at improving breastfeeding practices. With an overwhelming majority of infants not exclusively breastfed, it suggests an urgent need to find innovative strategies to reach mothers and key family members residing in these settings on benefits associated with exclusive breastfeeding.

In our analysis, we found educated mothers were more likely to discontinue exclusively breastfeeding than non-educated mothers. These findings were in contrast with evidence from developed countries [1], where the odds of complying with breastfeeding recommendations are higher among educated mothers compared with less educated mothers. While it is not clear why this is the case, this pattern supports findings from similar study in Nepal [16] and other developing country settings [17] where the prevalence of exclusive breastfeeding was higher among illiterate mothers. It is likely that educated mothers are more exposed to breastmilk substitutes and might perceive them as a viable modern alternative [18]. Poor breastfeeding practices among educated mothers in this setting reflect a poor knowledge and lack of accessible educational opportunities on the best feeding practices. Moreover, if breastfeeding promotional activities are targeted at educated women, then they can play a key role in spreading the message across society.

Mothers from the pucca households were more likely to cease exclusively breastfeed than those from the mixed/kutcha households. Household type could reflect the long-term socio-economic status of a family and studies from low and middle income countries (LMIC) report a similar higher prevalence of exclusive breastfeeding in lower socio-economic groups [19, 20]. The number of children in the family is found to be negatively associated with the duration of exclusive breastfeeding. Similar results have been reported that mothers having more than one child, were more likely to cease to exclusively breastfeed their infants during the first six months [21]. As the presence of other children might contribute towards maternal fatigue, it is important to target this group in breastfeeding promotion and support programmes. The early cessation of exclusive breastfeeding among mothers in a joint family compared to those living in nuclear or extended family needs further investigation to understand the socio-cultural factors underlying the family structures and breastfeeding practices [22]. We found a significant relationship between the season of a birth and exclusive breastfeeding: infants born during summer were more likely to stop exclusive breastfeeding compared to those born during winter months. Our results are in agreement with studies from India [23] and Europe [24] where children born during summer were breastfed for a shorter period of time. However, results from African studies [25, 26] are in contrast to our findings. This could be explained by the strong presence of hunger (rainy months) and harvest (hot months) seasons in African settings. In South Asian tropical climates, summer is the pre-harvesting season and hence likely to be associated with a lack of adequate maternal nutrition. Therefore, our results could reflect mothers’ concern about their infants’ lack of adequate hydration during summer months prompting them to supplement with liquid or semi-solid foods.

The major strength of the study was the prospective design which helped the assessment of exclusive breastfeeding since birth more accurately than cross-sectional or retrospective studies. In cross-sectional or retrospective studies, estimates of exclusive breastfeeding rates are based on maternal recall which often tends to overestimate the duration of exclusive breastfeeding, leading to biased measures of association [16]. Further, pooling data from three similar birth cohort studies resulted in larger sample size of 1, 088, reducing the risk of chance findings and adding statistical power to the analyses of relevant determinants. With rigorous follow-up, information for most children is available and hence reduces the bias due to attrition.

The main limitation of this study is that it was not designed to assess the determinants of exclusive breastfeeding. A study designed to identify key factors associated with exclusive breastfeeding would likely include additional relevant factors that were not collected as a part of the existing studies. Also, missing information on antenatal visits, prelacteal feeding, time of initiation of breastfeeding, maternal nutrition, and vaccination schedule did not allow us to investigate these factors. Additionally, the studies did not collect information on formula feeding, animal milk feeding, support from family and health workers, and the reasons for discontinuing exclusive breastfeeding.

## Conclusions

The practice of exclusive breastfeeding is considerably low among mothers living in urban slums settlements. Given the consequences of poor breastfeeding practices, there is a need to evaluate interventions educating the importance of breastfeeding to mothers and their families in reducing the gap between breastfeeding recommendations and practices.

## Abbreviations

AHR: Adjusted Hazard Ratio
CI: Confidence interval
LMIC: Low and middle income countries
